# Nanohydroxyapatite/Peptide Composite Coatings on Pure Titanium Surfaces with Nanonetwork Structures Using Oyster Shells

**DOI:** 10.3390/nano14070577

**Published:** 2024-03-26

**Authors:** Kuan-Hsiang Hsieh, Hsueh-Chuan Hsu, Yu-Lin Kao, Shih-Ching Wu, Tzu-Yen Yang, Wen-Fu Ho

**Affiliations:** 1 Department of Surgery, Division of Orthopaedics, Zuoying Armed Forces General Hospital, Kaohsiung 813204, Taiwan; bedbedegg2013@gmail.com; 2 Department of Dental Technology and Materials Science, Central Taiwan University of Science and Technology, Taichung 406053, Taiwan; hchsu@ctust.edu.tw (H.-C.H.); scwu@ctust.edu.tw (S.-C.W.); 3 Department of Life Sciences, National University of Kaohsiung, Kaohsiung 811726, Taiwan; yulinkao@nuk.edu.tw; 4 Department of Chemical and Materials Engineering, National University of Kaohsiung, Kaohsiung 811726, Taiwan

**Keywords:** titanium, hydrothermal synthesis, oyster shells, hydroxyapatite, peptides

## Abstract

Titanium and its alloys are extensively applied in artificial tooth roots because of their excellent corrosion resistance, high specific strength, and low elastic modulus. However, because of their biological inertness, their surface needs to be modified to improve the osteointegration of titanium implants. The preparation of biologically active calcium–phosphorus coatings on the surface of an implant is one effective method for enhancing the likelihood of bone integration. In this study, osteoinductive peptides were extracted from oyster shells by using acetic acid. Two peptide-containing hydroxyapatite (HA) composite coatings were then prepared: one coating was prepared by hydrothermally synthesizing an HA coating in the presence of peptides (HA/P/M), and the other coating was prepared by hydrothermally synthesizing HA and then immersing the hydrothermally synthesized HA in a peptide solution (HA/P/S). Characterization results indicated that the composite HA coatings containing oyster shell-based peptides were successfully prepared on the alkali-treated pure titanium surfaces. The HA/P/M and HA/P/S composite coatings were found to exhibit excellent hydrophilicity. Protein adsorption tests confirmed that the HA/P/M and HA/P/S coatings had an approximately 2.3 times higher concentration of adsorbed proteins than the pure HA coating.

## 1. Introduction

Titanium and its alloys are used as biomedical implants owing to their high specific strength, low elastic modulus, and excellent corrosion resistance [1,2]. However, they are bioinert and cannot bond with living bone; thus, surface modification is necessary. Surface chemical treatment is a common modification method; acid treatment [3], alkali treatment [4], and anodization [5] are various types of surface chemical treatments. In addition, bioactive ceramic coatings, such as hydroxyapatite [Ca_10_(PO_4_)_6_(OH)_2_; denoted HA], can be prepared on the surface of a bioinert material. HA, which is the key inorganic component of human bone and teeth, exhibits excellent biocompatibility and effectively improves the integration of implants with surrounding bone [6].

Plasma spray, electrochemical deposition, ultrasonic coating, and sol–gel techniques are commonly used for coating preparation [7,8,9]. Coatings are typically subjected to heat treatment to enhance their strength and crystallinity; however, high temperatures may affect the structure and performance of the underlying metal substrate. Hydrothermal reaction is an established method for preparing a uniform and dense HA coating [10]; it can promote recrystallization of the HA coating at relatively low temperatures [11]. In addition, before hydrothermal synthesis is performed, alkali treatment can be used to enhance the bonding of the coating to titanium metal. After alkali treatment, a nanoporous sodium titanate film develops on the surface of titanium, creating a negatively charged surface that promotes the crystallization of HA from a solution containing calcium and phosphate. Furthermore, the nanoporous film enhances the surface roughness and the adhesion properties of the HA coating [12].

The investigation of composite materials comprising HA and organic compounds (collagenous proteins [13], amino acids [14], chitosan [15], and polypeptides [16]) represents a promising research direction; these materials exhibit excellent bioactivity and can help regulate the growth and resorption of bone mineral crystals. Kojima et al. [17] indicated that peptides can combine with HA to form a peptide-HA composite and can modify the characteristics of HA particles, such as their surface potential, to improve protein adsorption through electrostatic interaction.

Recently, there has been a significant increase in research focused on the development of surface modifications with antimicrobial properties. In a study by Predoi et al. [18], zinc-doped HA coatings were developed on silicon substrates using a sol–gel spin-coating method. The zinc-doped HA layers demonstrated antimicrobial efficacy against *S. aureus* and *C. albicans*, underscoring their potential for applications demanding antimicrobial surfaces. Also, Predoi et al. [19] introduced the innovative development and analysis of thin films based on HA and silver-doped HA, enhanced with the antibiotics tetracycline and ciprofloxacin, aimed at combating the rise in antimicrobial resistance. The antibacterial efficacy of ciprofloxacin and tetracycline was significantly enhanced when applied in conjunction with HA and silver-doped HA layers against harmful bacteria strains, such as *Staphylococcus aureus* and *Escherichia coli*, underscoring the potential of these composite materials in medical and dental applications to prevent bacterial infections.

Many studies have been conducted on antimicrobial marine peptides. Marine organisms use antimicrobial peptides to prevent the invasion of pathogens and bacteria [20]. Antimicrobial peptides can become adsorbed on negatively charged bacteria because most antimicrobial peptides are positively charged. Moreover, these peptides are both hydrophobic and hydrophilic, which can lead to an imbalance in osmotic pressure due to the cell membrane permeabilization of bacteria [21,22]. Oyster shells have been confirmed to contain antimicrobial peptides and have antibacterial activity against various Gram-negative and Gram-positive bacteria [23,24].

An oyster shell is a biological composite material comprising >95% calcium carbonate and <5% organic matter. The organic matter—which includes proteins, glycoproteins, chitin, and polysaccharides—plays a crucial role in biomineralization [25]. An oyster shell contains a water-soluble matrix, which exhibits osteoinductive and antibacterial characteristics [26,27,28,29].

In this study, oyster shell powder was used as a raw material. Acetic acid was used to extract oyster shell-based peptides from the powder. The remaining powder was then used to synthesize HA. Next, a hydrothermal reaction was performed to prepare an HA coating containing oyster shell-based peptides on the surface of alkali-treated pure titanium. To the best of our knowledge, this study is the first investigation regarding the preparation of an HA coating containing oyster shell-based peptides on the surface of pure titanium, where oyster shells were used as raw materials.

## 2. Materials and Methods

### 2.1. Materials

The oxide layer on the surfaces of pure titanium specimens (99.9% purity; 12 mm in diameter; 0.5 mm in thickness) was removed with #100, #400, #600, and #800 sandpaper. Subsequently, they were immersed in a 5 M NaOH solution at 60 °C for 24 h. Afterward, the specimens were cleaned twice ultrasonically in deionized water for 5 min each time and then air-dried at 45 °C for 24 h.

Next, 20 g of oyster shell powder was added to 100 mL of 10% (*v*/*v*) acetic acid aqueous solution. The mixture was stirred at 350 rpm and 4 °C for 24 h and then centrifuged at 10,000 rpm and 4 °C to separate the supernatant from the precipitate. The supernatant was subjected to dialysis purification to obtain a peptide solution. The synthetic oyster shell-based peptides were lyophilized (FD-5030, Panchum, Kaohsiung, Taiwan) and stored in a freezer at −20 °C until future use. The precipitate was washed with deionized water and subsequently dried at 45 °C for 7 days, after which it was calcined in a muffle furnace (CWF1200, Carbolite, Sheffield, UK) at 850 °C for 1 h, with the heating rate maintained at 1.5 °C/min. The final calcined product, comprising CaO, was used as a raw material for subsequent preparation of the HA coating.

Afterward, 4.26 g of the CaO powder was added to 40 mL of deionized water, which was then stirred with a magnetic stirrer at 300 rpm and 50 °C for 6.5 h. Next, an (NH_4_)_2_HPO_4_ solution [5.952 g of (NH_4_)_2_HPO_4_; 30 mL of deionized water] was added so that the calcium–phosphorus ratio was 1.67; the solution was subsequently stirred again at 300 rpm for 30 min. Finally, the solution’s pH was adjusted to 10 by using ammonia, and the solution was used for subsequent preparation of an HA coating on the surface of pure titanium.

### 2.2. Preparation of HA/Peptide-Based Composite Coatings

The synthesis and coating method utilized in this study is an original approach developed by the authors. Two processes were employed to prepare HA/peptide-based composite coatings. In the first method, the oyster shell-based peptides were added to the aforementioned solution containing calcium and phosphorus, and a 24 h hydrothermal reaction was conducted at 200 °C to prepare an HA/peptide coating on the surface of the alkali-treated pure titanium. Coatings prepared using this method are hereafter referred to as HA/P/M coatings. The second method was based on a two-step synthesis technique. First, the alkali-treated pure titanium surface was immersed in the solution containing calcium and phosphorus, and an HA coating was prepared through a 24 h hydrothermal reaction at 200 °C. Afterward, the HA-coated sample was immersed in the peptide solution at 25 °C for 24 h, after which it was rinsed with phosphate-buffered solution (PBS) and deionized water. Coatings prepared using this method are hereafter referred to as HA/P/S coatings.

### 2.3. Characterization

#### 2.3.1. XRD

A high-resolution X-ray diffractometer (HR-XRD) device (D8 Discover, Bruker, Yokohama, Japan) was used to analyze the crystalline phase of the coating and Cu–Kα (λ = 1.5405 Å) was generated using a Cu target; the operating voltage was 40 kV and operating current was 40 mA. Low-angle diffraction patterns of the coatings were obtained in the 2θ range 10°–60° with a scan speed of 2°/min, a step size of 0.02°, and an incidence angle of 0.5°.

#### 2.3.2. SEM

To examine the morphology of a coating’s surface, the surface was coated with platinum (current: 10 mA; sputtering time: 30 s) and then observed using a field emission scanning electron microscope (FE-SEM; JSM-6330TF, JEOL, Tokyo, Japan).

#### 2.3.3. FTIR

The characteristic functional groups of the coating were analyzed using a Fourier-transform infrared (FTIR) spectrometer (Cary 630, Agilent, Santa Clara, CA, USA) with a spectral resolution of 8 cm^−1^ and with 128 scans over the range 650–4000 cm^−1^. In addition, the KBr pellet technique was used to transform the peptide powder into a translucent sheet for transmission analysis.

#### 2.3.4. XPS

The composition of the coating layer was analyzed through X-ray photoelectron spectroscopy (XPS; JAMP-9500F, JEOL, Japan).

### 2.4. Contact Angle Measurement

The sample was sequentially cleaned with ethanol and deionized water. Once dried, it was affixed onto a 3D manipulator and aligned horizontally, and the camera was adjusted to the same orientation. Water contact angles were measured utilizing the sessile drop method, employing a contact angle meter (model 100SL, Sindatek, Taipei City, Taiwan) with a 1 µL volume of deionized water. Following each measurement, the sample was dried with tissue paper, and subsequent tests were conducted after a minimum interval of 5 min. Measurements were taken at five different locations on three separate samples for each sample type, and the average value, along with the standard deviation, was calculated. All testing procedures were conducted in accordance with the pertinent normative standards specified in ASTM D-5725 [30].

### 2.5. Protein Absorption Test

The samples were placed in a 24-well dish and sterilized under an ultraviolet lamp for 2 h. Next, 1 mL of minimum essential medium (MEM) containing 1% (*v*/*v*) fetal bovine serum (FBS) was added to each well, and the samples were soaked for 24 h. This process was conducted in an incubator at 37 °C. Subsequently, the samples were gently washed with PBS three times to remove nonsticky and loosely adhering proteins on the surface and then dried at 37 °C for 1 h. Afterward, the samples were immersed in 1% sodium dodecyl sulfate solution for 30 min to elute the proteins adsorbed on their surfaces. Finally, the proteins’ absorbance was measured at a wavelength of 750 nm by using a spectrophotometer (PowerWave HT 340, Biotek, Phoenix, AZ, USA). A calibration curve was established on the basis of a series of concentrations of standard bovine serum albumin (BSA), and a linear regression formula of this calibration curve was derived. Substituting the obtained absorbance value into the linear regression formula yielded the amount of protein adsorbed on the coating layer. Experiments were conducted using three independent samples for each sample type.

### 2.6. Statistical Analysis

All data in this study are presented as mean ± standard deviation. Statistical analyses for the results obtained from contact angle measurements and protein absorption tests were conducted using one-way analysis of variance (ANOVA), followed by Tukey’s test. Statistical significance was considered at *p* < 0.05.

## 3. Results and Discussion

### 3.1. Morphology

**Figure 1:**
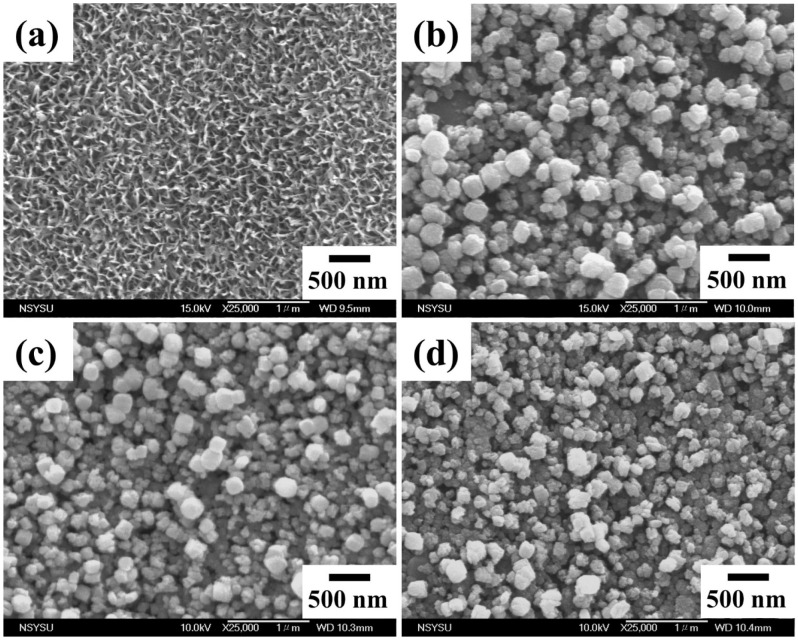
SEM images of pure titanium (**a**) after alkali treatment and with (**b**) an HA coating prepared through hydrothermal treatment; (**c**) an HA/peptide composite coating prepared through hydrothermal treatment (HA/P/M); and (**d**) an HA/peptide composite coating prepared through hydrothermal and subsequent soaking treatment (HA/P/S).

Figure 1 presents FE-SEM images of the pure titanium after alkali treatment and various subsequent surface coating treatments. After alkali treatment (5 M NaOH solution at 60 °C), the pure titanium surface exhibited a nanonetwork structure (Figure 1a); nanoscale roughness was observed on the titanium surface. In this study, the alkali-treated samples were immersed in a solution that contained Ca and P and was prepared from oyster shells; a 24 h hydrothermal reaction was then conducted at 200 °C. The nanonetwork structure of the samples treated in this manner was completely covered with spherical nanoparticles (Figure 1b). In addition, dense spherical nanoparticles were found to be present on the HA/peptide composite coatings prepared using the two investigated processes (Figure 1c,d). In the composite coating prepared through hydrothermal reaction, the HA took the form of spherical nanoparticles. The particle size ranges of the coatings for HA, HA/P/M, and HA/P/S (as shown in Figure 1b, c, and d) were 94–377, 132–321, and 132–377 nm, respectively, with average particle sizes of 238, 219, and 232 nm, respectively.

Generally, the pH values of Ca and P solutions affect the morphology of synthetic HA powder. An et al. [31] hydrothermally synthesized HA powder and found that when NaOH was added to increase the pH of the reaction solution from 10.5 to 12.5, the morphology of the synthesized HA particles changed from nanorods to spherical particles. In the present study, the pH of the reaction solution used to prepare the HA coating was maintained at 10; thus, other factors may influence the morphology of HA, and this topic needs further investigation in the future.

### 3.2. Phase Composition

**Figure 2:**
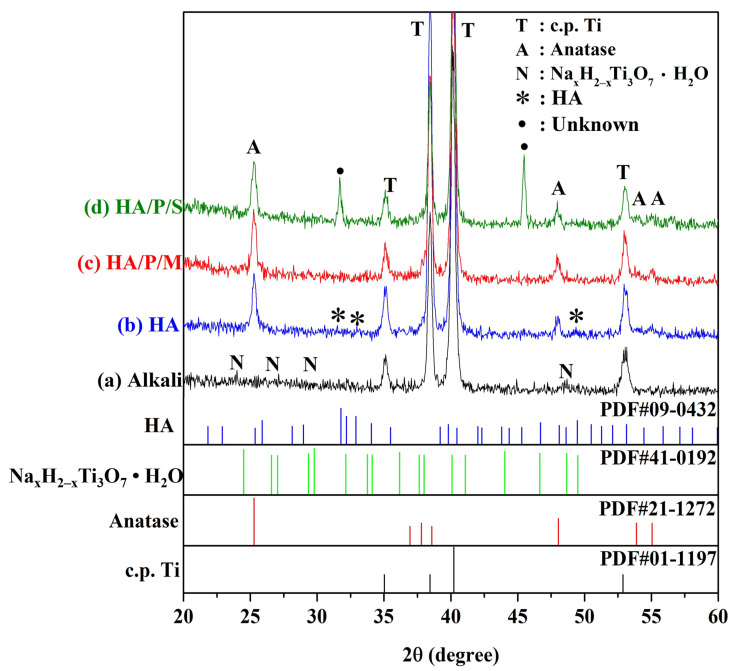
XRD patterns of pure titanium after alkali treatment and various types of surface-coating treatment.

Figure 2 shows the HR-XRD patterns of the pure titanium after surface treatment. The spectrum of the surface of the pure titanium treated with 5 M NaOH at 60 °C for 24 h contains diffraction peaks corresponding to the sodium titanate hydrogel (Na_x_H_2−x_Ti_3_O_7_H_2_O; Figure 2a). The alkali-treated samples were immersed in a solution containing Ca and P prepared from oyster shell powder and then subjected to hydrothermal treatment at 200 °C for 24 h. The XRD results (Figure 2c,d) indicated that in the HA/P/M and HA/P/S composite coatings, the sodium titanate hydrogel was transformed into a bioactive TiO_2_ anatase phase [32]. In the HA/P/S sample, two peaks remain unidentified and require further analysis. However, the HA peaks were not clearly observed because the coating was insufficiently thick, and the two types of HA/peptide coatings contained amorphous peptide molecules. Kojima et al. [17] found that the diffraction peaks in the spectrum of a peptide/HA composite were broader than those in the spectrum of pure HA and that their synthesized composite contained HA with low crystallinity. Moreover, Krukowski et al. [33] discovered that their glycine/HA composite contained HA with decreased crystallinity and a decreased number of hydroxyl groups because of the presence of glycine. In a study [15] investigating the fabrication of HA/chitosan composite coatings on Ti alloy surfaces, it was observed that HA displayed weaker and broader diffraction peaks, potentially attributed to the thinner and nanocrystalline nature of the HA coating.

**Figure 3:**
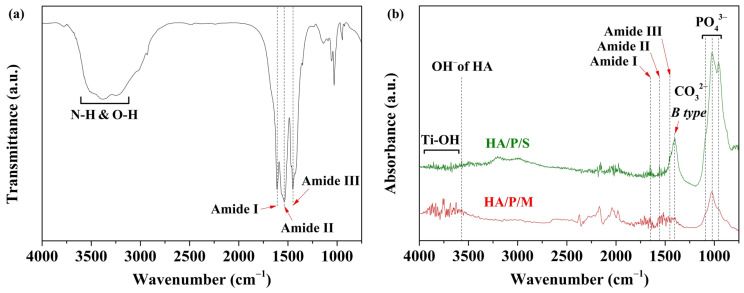
(**a**) FTIR spectrum of peptides extracted from oyster shells, and (**b**) FTIR–attenuated total reflectance spectra of HA/P/S and HA/P/M composite coatings prepared on pure titanium surfaces.

Figure 3a shows the FTIR spectrum of the peptide obtained by extracting oyster shell powder with acetic acid. Peptide molecules are small fragments of protein comprising amino acid monomers linked with amide bonds. Amide groups are key functional groups of peptide molecules; C=O, C–N, and N–H are such functional groups [17]. The peaks at 1610, 1538, and 1450 cm^−1^ correspond to amide I (C=O stretching vibration), amide II (C–N stretching vibration and N–H bending vibration), and amide III (C–N stretching vibration and N–H bending vibration), respectively. The wavelength range 3100–3700 cm^−1^ corresponds to the N–H and O–H stretching vibration of adsorbed water.

To confirm that HA and oyster shell-based peptides were prepared on the pure titanium surfaces of the samples, two HA/peptide composite coatings were subjected to FTIR analysis (Figure 3b). A comparison of the FTIR spectra presented in Figure 3a,b reveals peaks characteristic of amide I and amide II in the HA/P/M and HA/P/S coatings. The characteristic peaks in the spectrum of the HA/P/M coating were stronger because the HA/P/M coating was obtained through a one-step hydrothermal synthesis process, which led to more peptide molecules being incorporated into the interior part of the coating. However, the HA/P/S coating may have had peptides on the surface of the HA coating. In addition, peaks corresponding to the phosphate functional group (PO_4_^3−^) of the HA were observed at 950, 1022, and 1091 cm^−1^, as shown in Figure 3b, and a peak corresponding to the OH^−^ extension vibration of the HA was observed at approximately 3571 cm^−1^. A peak corresponding to B-type carbonate was present at 1403 cm^−1^, which indicated the substitution of CO_3_^2−^ for PO_4_^3−^ in the apatite lattice. Studies have shown that the properties of carbonate-containing HA are similar to those of human bone [34]; this biosimilarity can aid the growth of bone cells. On the basis of these results, the preparation of a bone-like HA coating containing oyster shell-based peptides on a pure titanium surface (HA/P/M and HA/P/S coatings) was concluded to have been successful; moreover, the HA/P/M process was found to have effectively incorporated more peptide molecules into the coating than was achieved using the HA/P/S process.

**Figure 4:**
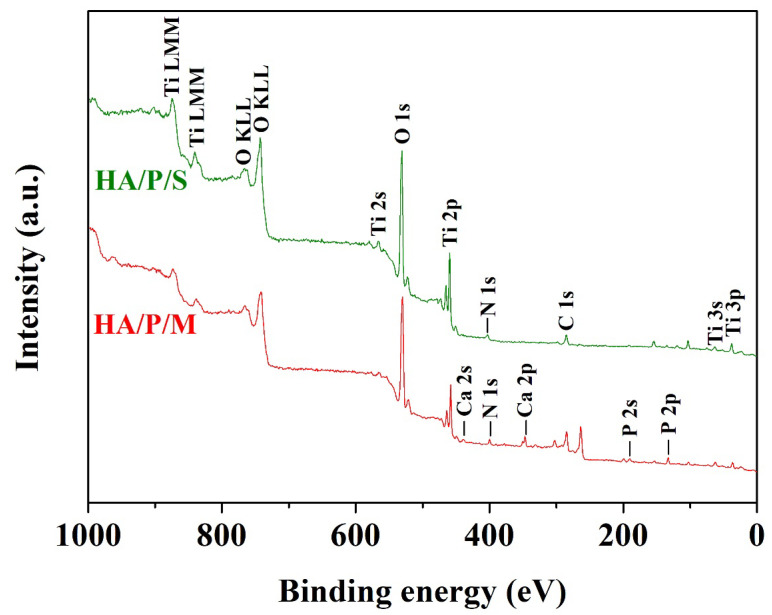
XPS survey spectra of HA/P/S and HA/P/M composite coatings prepared on pure titanium surfaces.

Figure 4 shows the XPS survey spectra of the HA/P/M and HA/P/S composite coatings. Nitrogen element analysis confirmed that peptide molecules existed in the coating. The binding energy of the N 1s peak was approximately 400 eV, corresponding to the amino groups and amide groups of the peptide molecules [35]. The signal corresponding to N confirmed the presence of peptide molecules in the HA/P/M coating. However, the N 1s binding energy of the HA/P/S coating was 401.5 eV, which corresponds to the ammonium ion (–NH^3+^); this may have been due to partial protonation of the amino group in the peptide molecules [36], and the peptides in the HA/P/S coating may not have been favorably immobilized.

**Figure 5:**
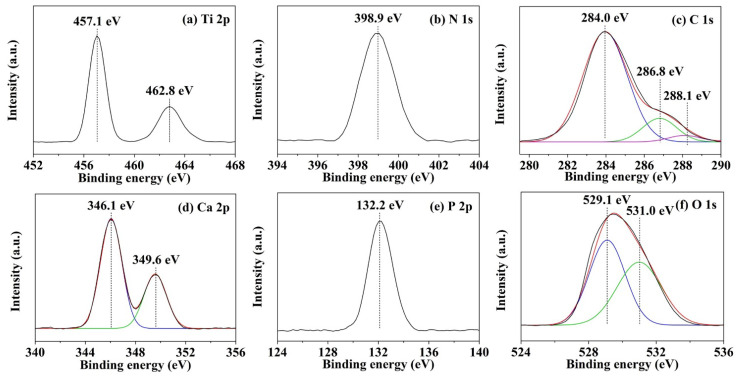
High-resolution XPS spectra of HA/P/M composite coating on a pure titanium surface.

Figure 5 displays the high-resolution spectra of the HA/P/M composite coating on the pure titanium surface. Figure 5a indicates that titanium on the surface of the substrate was mainly in the form of TiO_2_. The binding energy values were 462.8 (Ti 2p_1/2_) and 457.1 (Ti 2p_3/2_) eV [32]. The binding energy of N 1s (398.9 eV) corresponded to the C–NH_2_ group of peptide molecules [35] (Figure 5b). Figure 5c shows that the binding energy values of 284.0, 286.8, and 288.1 eV corresponded to C–C and C–H, C–O, and C=O bonds, respectively, which are characteristic carbon bonds of peptide molecules [37]. The characteristic peak at 284.0 eV confirmed the presence of carbonate ions in the HA structure [38]. The Ca 2p peaks (Ca 2p_1/2_ at 349.6 eV and Ca 2p_3/2_ at 346.1 eV) and the P 2p peak (132.2 eV) confirmed the presence of calcium phosphate in the HA/P/M coating (Figure 5d,e). Furthermore, the O 1s peak at 531.0 eV (Figure 5f) and the P 2p peak at 132.2 eV (Figure 5e) confirmed that the calcium phosphate in the coating was in the form of HA [32]. The oxygen peak at 529.1 eV corresponded to the O 1s binding in TiO_2_ [32]. Therefore, the XPS results confirmed that an HA composite coating containing oyster shell-based peptides, obtained through alkali treatment and subsequent one-step hydrothermal synthesis, had been successfully prepared on the pure titanium surface.

### 3.3. Wettability

**Figure 6:**
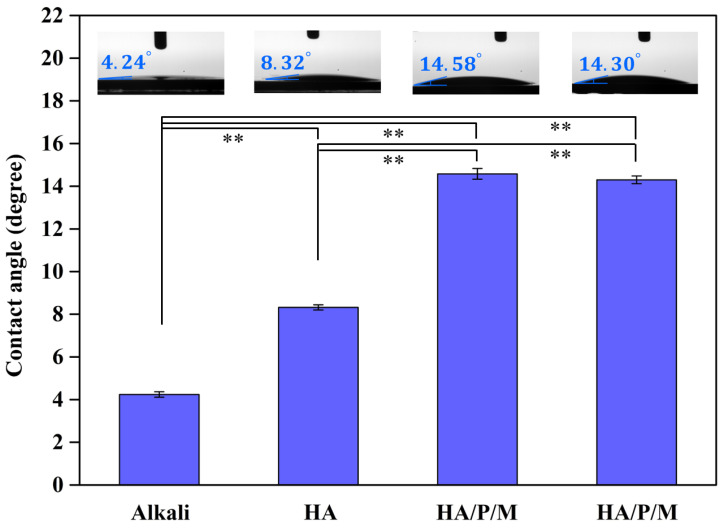
Contact angles of the surfaces of pure titanium after alkali treatment and various types of surface coating treatment. Statistical significance (**) was set at a *p*-value of less than 0.01.

Through contact angle measurements of water droplets, the wettability of the pure titanium samples after surface treatment was evaluated (Figure 6). The contact angle of the pure titanium surface after alkali treatment was 4.24°, indicating superhydrophilicity. The contact angle of the pure titanium surface prepared through alkali treatment and subsequently given an HA coating was 8.32°. Because the Ca and P ions in the solution reacted with hydroxide on the surface of the alkali-treated sample, the concentration of hydroxides (hydrophilic groups) was reduced; consequently, the level of hydrophilicity of the HA coating’s surface was significantly lower (*p* < 0.01) than that of the alkali-treated surface. In addition, the HA/P/M and HA/P/S composite coatings had similar contact angles, that is, 14.58° and 14.30°, respectively; both surfaces were, thus, hydrophilic. The significantly greater (*p* < 0.01) contact angles of the HA/P/M and HA/P/S coatings compared with the other samples were attributable to the amino acid and hydrophobic amino acid residues of peptide molecules [39,40]. The wettability of an implant’s surface influences the adsorption of proteins and cells in the human environment. Therefore, implants with a small contact angle exhibit excellent blood wettability, cell adhesion, and affinity for protein adsorption and are, thus, more likely to undergo osseointegration [41]. However, a large number of water molecules accumulate on the surface of a superhydrophilic coating, making it difficult for proteins to pass through tightly bound water molecules and, thus, hindering protein adsorption [42].

### 3.4. Protein Absorption Test

**Figure 7:**
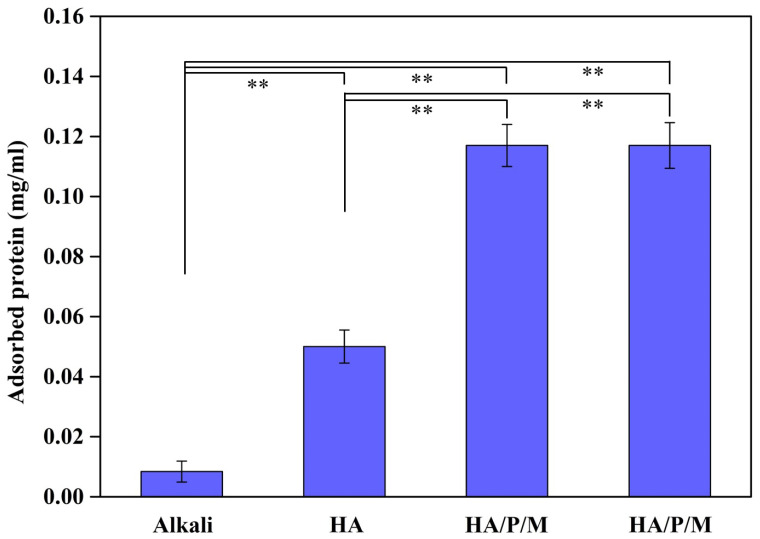
Protein adsorption capacities of the surfaces of pure titanium after alkali treatment and various types of surface coating treatment. The samples were cultured for 24 h in MEM containing 1% FBS. Statistical significance (**) was set at a *p*-value of less than 0.01.

Alkali-treated pure titanium, an HA-coated titanium sample, and two HA/peptide composite coatings (HA/P/M and HA/P/S) were soaked in MEM containing 1% FBS and cultivated for 24 h to evaluate their protein adsorption capacity (Figure 7). The concentration of proteins on the alkali-treated surface was 0.00917 mg/mL, and that on the HA-coated titanium sample was 0.05083 mg/mL, confirming that the HA-coated surface had significantly greater (*p* < 0.01) protein adsorption capacity than the alkali treatment surface. The HA/P/M and HA/P/S composite coatings had even greater protein adsorption capacity; the corresponding protein concentration was 0.1175 mg/mL (2.3 times significantly higher (*p* < 0.01) than that of the HA-coated sample). Silva-Bermudez and Rodil [43] argued that protein adsorption capacity is not only related to the wettability of the coating’s surface but also affected by the electrostatic interaction between the coating surface and proteins. In the present study, the HA/P/M and HA/P/S composite coatings exhibited greater protein adsorption capacity because the incorporation of peptide molecules led to a change in the surface static charge of the HA coating and an increase in the electrostatic attraction between the coating surface and proteins. Because effective protein adsorption can promote cell adhesion and proliferation, protein adsorption capacity can be used as an initial indicator of the surface biocompatibility of a metal implant [43].

## 4. Conclusions

In this study, peptides were successfully extracted from oyster shells. In addition, HA coatings containing oyster shell peptides were prepared on alkali-treated pure titanium surfaces through two processes, HA/P/M and HA/P/S. The findings of this study are summarized as follows:

1. When pure titanium was treated with a 5 M NaOH solution at 60 °C for 24 h, a nanonetwork structure formed on the pure titanium surface. The HA on the surfaces of the HA/P/M and HA/P/S composite coatings were spherical nanoparticles.
2. The XRD results indicated that the HA/P/M and HA/P/S composite coatings exhibited the presence of bioactive TiO_2_ anatase along with low crystallinity HA.
3. FTIR analysis showed that the peptides extracted from oyster shells included characteristic amide I, II, and III functional groups.
4. The HA coating was synthesized using the oyster shells, and The HA coating contained B-type carbonate; the structure of the B-type carbonate was similar to the inorganic mineral structure of human bone.
5. The static contact angle measurements indicated that HA/P/M (14.58°) and HA/P/S (14.30°) exhibited excellent surface wettability.
6. The results of the protein adsorption test revealed that the HA composite coating containing oyster shell peptides had an approximately 2.3 times higher concentration of adsorbed protein than the pure HA coating. The oyster shell peptides helped boost the adsorption of protein on the coatings’ surface, thereby enhancing the coatings’ biocompatibility. Hence, the HA/P/M and HA/P/S composite coatings fabricated on Ti surfaces in this study hold promise for potential applications as dental implants.

## Data Availability

Data are contained within the article.
